# Interpolative multidimensional scaling techniques for the identification of clusters in very large sequence sets

**DOI:** 10.1186/1471-2105-13-S2-S9

**Published:** 2012-03-13

**Authors:** Adam Hughes, Yang Ruan, Saliya Ekanayake, Seung-Hee Bae, Qunfeng Dong, Mina Rho, Judy Qiu, Geoffrey Fox

**Affiliations:** 1 Pervasive Technology Institute, Indiana University, Bloomington, IN 47408, USA; 2 School of Informatics and Computing, Indiana University, Bloomington, IN 47408, USA; 3 Department of Biological Sciences, University of North Texas, Denton, TX 76203, USA

## Abstract

**Background:**

Modern pyrosequencing techniques make it possible to study complex bacterial populations, such as *16S rRNA*, directly from environmental or clinical samples without the need for laboratory purification. Alignment of sequences across the resultant large data sets (100,000+ sequences) is of particular interest for the purpose of identifying potential gene clusters and families, but such analysis represents a daunting computational task. The aim of this work is the development of an efficient pipeline for the clustering of large sequence read sets.

**Methods:**

Pairwise alignment techniques are used here to calculate genetic distances between sequence pairs. These methods are pleasingly parallel and have been shown to more accurately reflect accurate genetic distances in highly variable regions of *rRNA *genes than do traditional multiple sequence alignment (MSA) approaches. By utilizing Needleman-Wunsch (NW) pairwise alignment in conjunction with novel implementations of interpolative multidimensional scaling (MDS), we have developed an effective method for visualizing massive biosequence data sets and quickly identifying potential gene clusters.

**Results:**

This study demonstrates the use of interpolative MDS to obtain clustering results that are qualitatively similar to those obtained through full MDS, but with substantial cost savings. In particular, the wall clock time required to cluster a set of 100,000 sequences has been reduced from seven hours to less than one hour through the use of interpolative MDS.

**Conclusions:**

Although work remains to be done in selecting the optimal training set size for interpolative MDS, substantial computational cost savings will allow us to cluster much larger sequence sets in the future.

## Background

The continued advancement of pyrosequencing techniques has made it possible for scientists to study complex bacterial populations, such as *16S rRNA*, directly from environmental or clinical samples without the need for involved and time-consuming laboratory purification [1]. As a result, there has been a rapid accumulation of raw sequence reads awaiting analysis in recent years, placing an extreme burden on existing software systems. Alignment of sequences across these large data sets (100,000+ sequences) is of particular interest for the purposes of sequence classification and identification of potential gene clusters and families, but such analysis cannot be completed manually and represents a daunting computational task. The aim of this work is the development of an efficient and effective pipeline for clustering large quantities of raw biosequence reads.

## Methods

One technique often used in sequence clustering is multiple sequence alignment (MSA), which employs heuristic methods in an attempt to determine optimal alignments across an entire sample. However, global pairwise sequence alignment algorithms have previously been reported to better identify microbial richness in genomes with hypervariable regions, like *16S rRNA*, than do MSA techniques, while also offering superior computational scaling [1]. For these studies, genetic distances produced by the Needleman-Wunsch pairwise aligner algorithm [2] were converted to Cartesian coordinates through Multidimensional Scaling (MDS) for the purpose of clustering and visualization [3].

**Figure 1:**
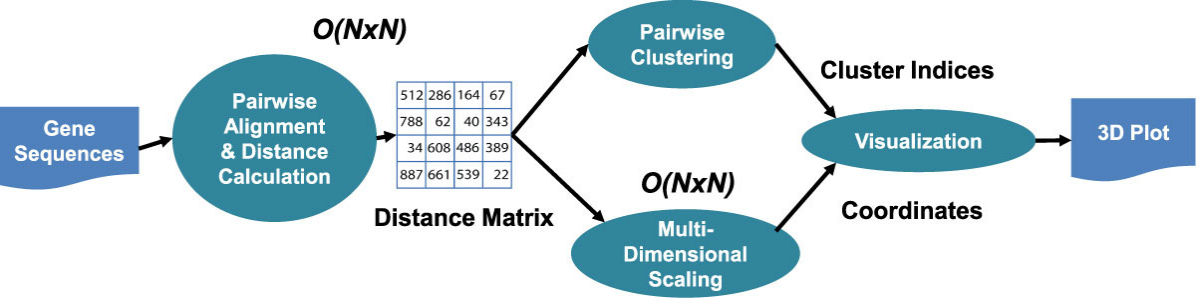
**Basic computational pipeline for sequence clustering**. Sequence clustering begins with a sampling of raw sequence reads, stripped of duplicates. Pairwise sequence alignments and genetic distances are calculated over the entire sample. For this study, the Needleman-Wunsch global alignment algorithm was employed. Next, the calculated distances are passed to multidimensional scaling and pairwise clustering algorithms, producing Cartesian coordinates and clustering information which can be used to visualize the sequence space. Both the distance calculation and multidimensional scaling are order *O*(N^2^), where N is the number of sequences, making the pipeline computationally expensive as the sample grows very large.

This basic clustering pipeline is shown in Figure 1, and it has been used to good effect for sample sizes of 100,000 or fewer sequences with less than 200 bases in each. However, the computational complexity of both the distance calculation and multidimensional scaling is *O*(N^2^), where N is the number of sequences, rendering the overall process untenable as the sample size grows very large.

**Figure 2:**
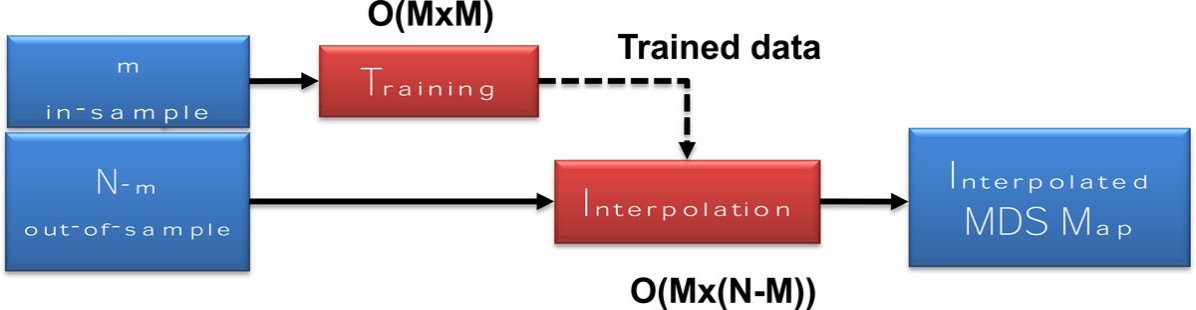
**Interpolative Multidimensional Scaling (MDS)**. Interpolative MDS begins with a raw sequence file, which is then divided into in-sample and out-of-sample sets. The in-sample data is then subjected to full NW distance and MDS calculations, resulting in a subset of genetic distances. This trained data is then used to interpolate the distances for the remaining, out-of-sample sequences. The computational complexity of the interpolation step is *O*((N-M)*M), where N is the size of the original sequence set and M is the size of the in-sample data.

To overcome these performance bottlenecks, we have employed an interpolative MDS algorithm [4], wherein a small, in-sample subset of sequences is subjected to full NW and MDS calculations and then the results are used to interpolate Cartesian coordinates for the remaining, out-of-sample sequences from the larger data set. This reduces the computational complexity to O(M^2^) + O(M*(N-M)) for both distance and scaling operations, where N is the number of sequences in the initial data set, M is the number of in-sample sequences, and N-M is the number of out-of-sample sequences. The basic interpolative MDS scheme is illustrated in Figure 2.

**Figure 3:**
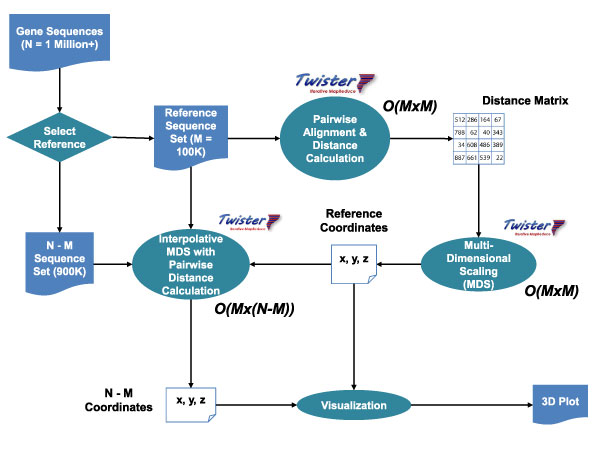
**Scaled-up computational pipeline for sequence clustering**. As with the basic pipeline, the scaled-up workflow begins with a raw sequence file. Before calculating genetic distances, the file is divided into in-sample and out-of-sample sets for use in Interpolative MDS. Full MDS and NW distance calculations on the in-sample data yield trained distances, which are used to interpolate the remaining distances. The interpolation step includes on-the-fly pairwise NW distance calculation. The overall complexity of the pipeline is reduced from *O*(N^2^) for the basic pipeline to *O*(M^2 ^+ (N-M)*M) for the pipeline with interpolation, where N is the size of the original sequence set and M is the size of the in-sample data. To enhance computational job management and resource availability, all computational portions of the depicted pipeline were implemented using the Twister Iterative Map Reduce runtime.

To further enhance computational throughput and ease job management, we implemented the updated pipeline utilizing the Twister Iterative MapReduce runtime [5] to take advantage of the map-reduce pattern inherent in these calculations. Twister, developed in our lab, also enables us to target large, Linux-based compute clusters [6]. This scaled-up pipeline is shown in Figure 3.

## Results and discussion

### Full calculation on entire data set

**Figure 4:**
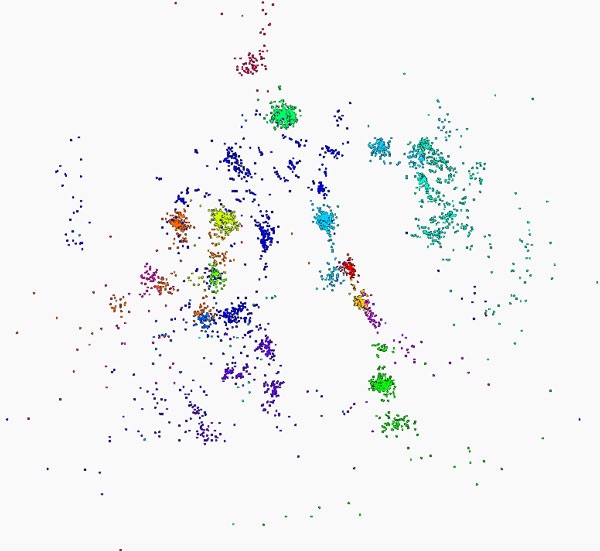
**100K Metagenomics sequences - Full MDS**. Visualization of MDS and clustering results for 100,000 gene sequences from an environmental sample of *16S rRNA*. The many different genes are classified by a clustering algorithm and visualized by MDS dimension reduction.

Figure 4 shows the results of running full Needleman-Wunsch (NW) and Multidimensional Scaling (MDS) calculations on a set of 100,000 raw *16S rRNA *sequence reads. The results of this calculation fit well with the expected groupings for this genome [7,8]. The initial clustering calculation colors the predicted sequences in a given grouping, while the MDS calculation produces Cartesian coordinates for each sequence. As Figure 4 shows, the spatial and colored results correspond to the same sequences, indicating that the combination of NW and MDS produce reasonable sequence clusters.

### Interpolation: 50000 in-sample sequences, 50000 out-of-sample sequences

**Figure 5:**
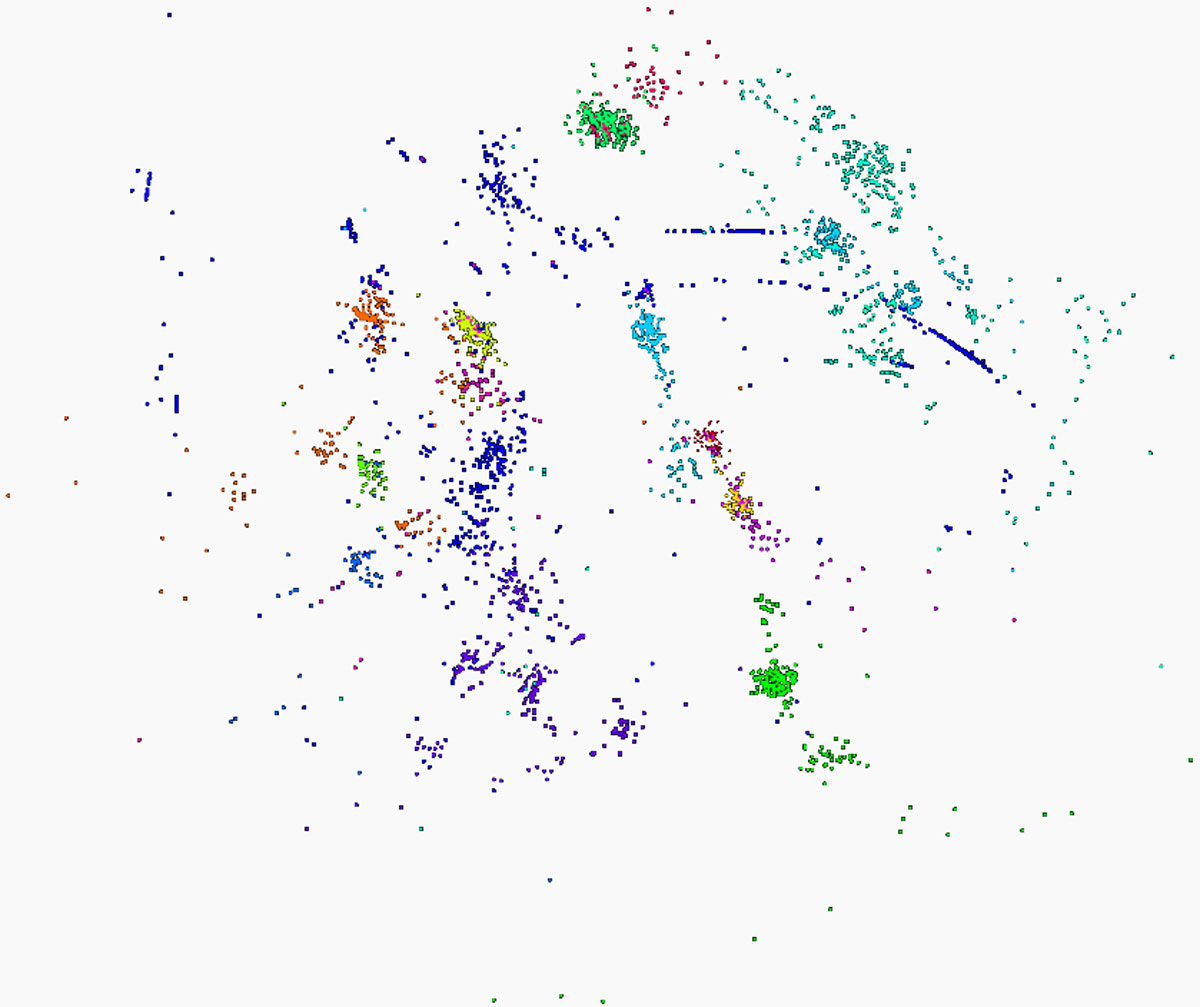
**100K Metagenomics sequences - 50K interpolated points**. Visualization of the same 100,000 gene sequences clustered by interpolative MDS. Half of the coordinate sets were generated by full MDS calculation, and the other 50,000 were interpolated from the in-sample results. The basic structure observed in the full MDS calculation can also be seen here.

Figure 5 shows the results of running interpolative MDS and NW on the same 100,000 sequences, with 50,000 in-sample and 50,000 out-of-sample data points. The basic structure observed in this case is similar to that seen in the full calculation discussed above. Some slight differences within individual clusters are noted, but the major sequence groupings are intact.

### Interpolation: 10000 in-sample sequences, 90000 out-of-sample sequences

**Figure 6:**
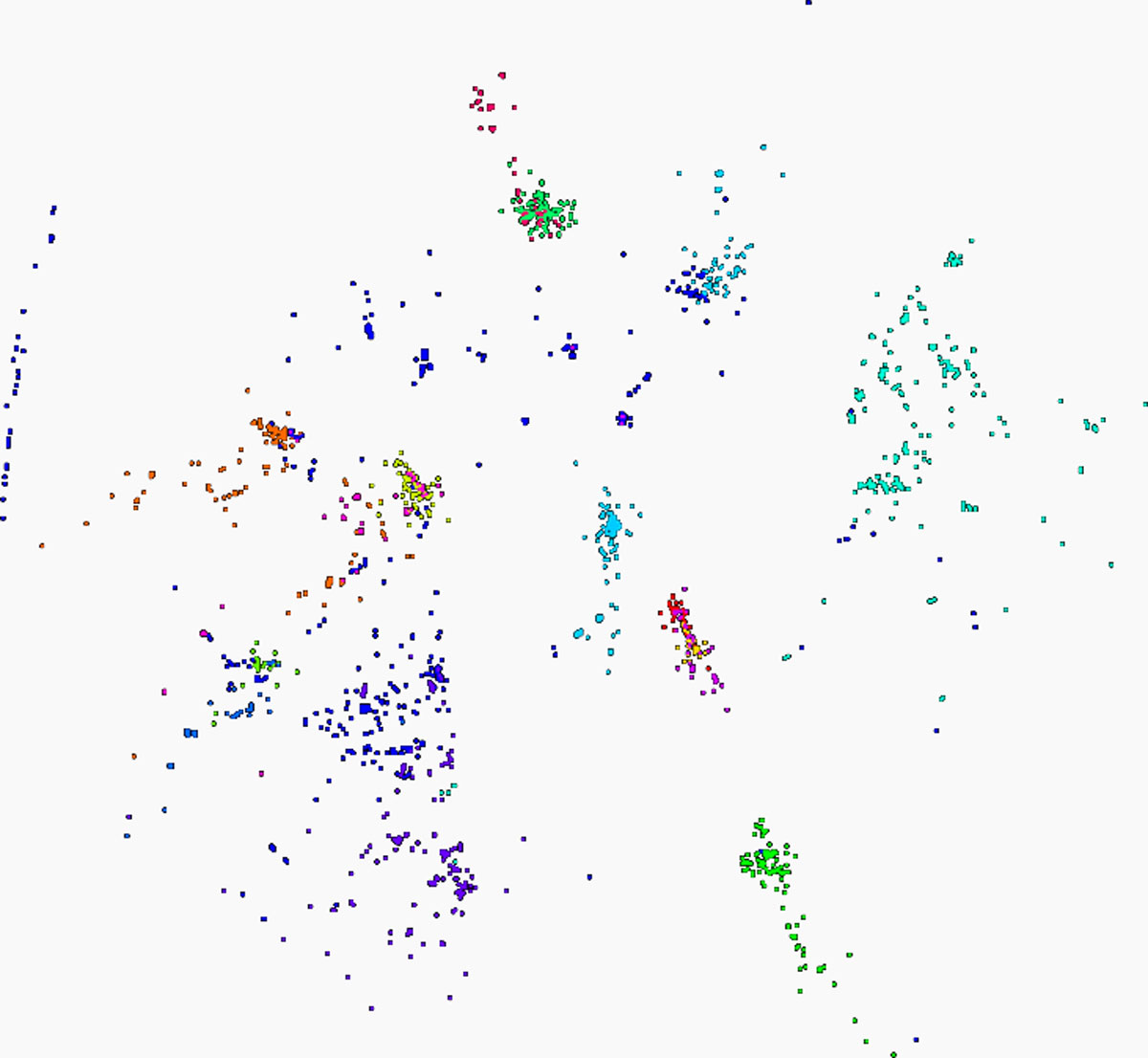
**100K Metagenomics sequences - 90K interpolated points**. Visualization of the same 100,000 gene sequences clustered by interpolative MDS. Ten thousand of the coordinate sets were generated by full MDS calculation, and the other 90,000 were interpolated from the in-sample results. The basic structure observed in the full MDS calculation can also be seen here, with some degradation of resolution within clusters.

Figure 6 shows the results of running interpolative MDS and NW on the same 100,000 sequences, with 10,000 in-sample and 90,000 out-of-sample data points. Once again, the same basic clustering structure is observed, although more significant changes in intra-cluster arrangement can be seen.

### Performance results

**Figure 7:**
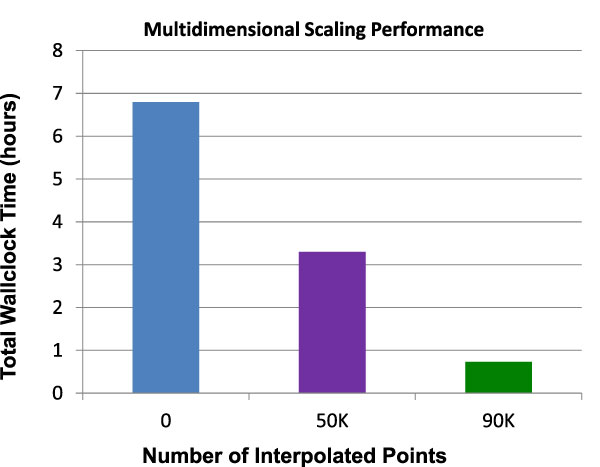
**Multidimensional Scaling performance**. Performance results for the three runs presented in this paper. Full MDS for the entire sample of 100,000 gene sequences required about seven hours to complete on 90 nodes (720 cores) of Polar Grid Quarry at Indiana University. Interpolation with equal in-sample and out-of-sample sizes (50,000 sequences each) required about three and half hours to complete. Interpolation with 10,000 in-sample sequences and 90,000 out-of-sample sequences required less than an hour to complete.

Figure 7 shows the wall-clock time required to run each complete pipeline discussed above. The full, non-interpolative calculation required about seven hours, while the interpolative pipeline consisting of 50,000 in-sample and 50,000 out-of-sample points required about three-and-a-half hours. Finally, the interpolative calculation with 10,000 in-sample and 90,000 out-of-sample sequences completed in a little under an hour.

## Conclusions

This study demonstrates the effectiveness of combining the Needleman-Wunsch genetic distance algorithm with Multidimensional Scaling (MDS) to enable visual identification of sequence clusters in a large sample of raw reads from the *16S rRNA *genome. In addition, the use of interpolative MDS and the Twister Iterative MapReduce runtime provides significant improvement in overall computational throughput while maintaining the basic structure of the resultant sequence space. Further investigation is needed to determine the optimal ratio of in-sample to out-of-sample data set sizes in order to strike the proper balance between performance and intra-cluster detail. Future plans include the study of other genomes and scaling up these studies to cluster millions of sequence reads in the span of a single pipeline run.

## List of abbreviations used

NW: Needleman-Wunsch; MDS: Multidimensional Scaling; MSA: Multisequence Alignment; rRNA: ribosomal ribonucleic acid.

## Competing interests

The authors declare that they have no competing interests.

## Authors' contributions

AH participated in software development and sequence clustering, and drafted the manuscript. YR, SE and SHB participated in software development and sequence clustering. QD provided sequence samples for study and participated in results analysis. MR provided sequence samples for study and participated in study design and results analysis. JQ leads the SALSA lab where this work was performed and provided guidance in the use of the Twister map-reduce runtime in the computational pipeline. GF conceived of the study, participated in its design and coordination, and helped refine the manuscript.
